# Oxidized regenerated cellulose combined with hyperbaric oxygen therapy successfully managed delayed bleeding after peroral endoscopic myotomy for achalasia

**DOI:** 10.1055/a-2366-1536

**Published:** 2024-09-26

**Authors:** Dingguo Zhang, Shenggang Zhan, Qiuling Lin, Zhiyuan Zou, Lisheng Wang

**Affiliations:** 1 Department of Gastroenterology, Shenzhen People’s Hospital, First Affiliated Hospital of Southern University of Science and Technology, Second Clinical Medical College of Jinan University, Shenzhen, China; 2 Department of General Practice, Shenzhen People’s Hospital, First Affiliated Hospital of Southern University of Science and Technology, Second Clinical Medical College of Jinan University, Shenzhen, China

A 35-year-old man diagnosed with type 1 achalasia underwent peroral endoscopic myotomy (POEM) successfully. Unfortunately, red blood was drained from the gastric tube 10 hours post-procedure.

An emergency gastroscopy revealed fresh red blood, swelling, and stenosis in the esophagus ( Fig. 1 **a** ). Attempts to establish a new tunnel for hemostasis were unsuccessful. The clips were removed, and a large number of blood clots were observed within the tunnel ( Fig. 1 **b** ), which made precise hemostasis impossible. Sengstaken–Blakemore tube compression achieved hemostasis temporarily, but rebleeding occurred after its removal 1 . On postoperative Day 4, a repeat gastroscopy indicated increased swelling and congestion in the esophageal cavity, with ongoing oozing within the tunnel. Hemostasis was then achieved by packing the tunnel with absorbable oxidized regenerated cellulose ( Fig. 1 **c** ) 2 . Subsequently, a gastric tube for negative pressure suction above the tunnel orifice and a jejunal tube for enteral nutrition were placed.

**Fig. 1:**
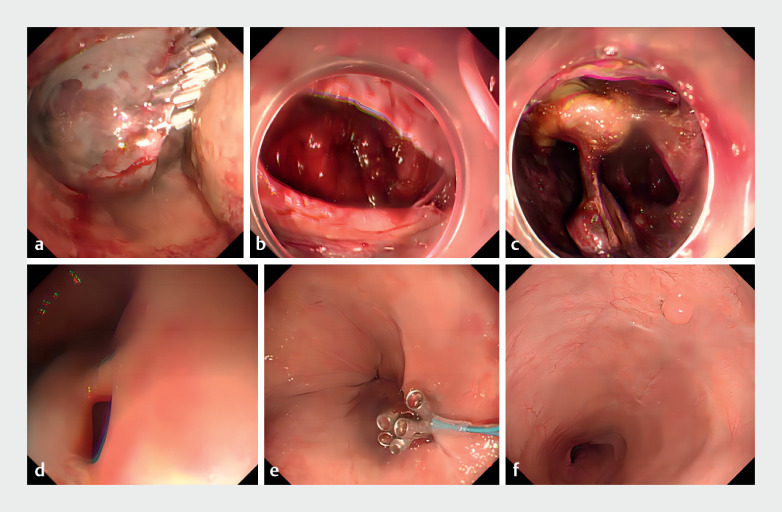
Endoscopic images. **a** Emergency gastroscopy revealed fresh red blood, swelling, congestion, and stenosis of the esophagus. **b** After removing the clips, a large number of blood clots were observed within the tunnel cavity. **c** On postoperative Day 4, a repeat gastroscopy indicated ongoing active oozing within the tunnel. The tunnel was then packed with absorbable oxidized regenerated cellulose. **d** Gastroscopy 2 weeks later showed successful closure of the original tunnel orifice, but the new tunnel orifice remained open. **e** Clips, purse-string sutures, and tissue adhesive application in succession were performed to close the new tunnel orifice. **f** Follow-up gastroscopy 2 months later confirmed complete wound healing.

Follow-up gastroscopy 2 weeks later showed improvement in esophageal edema, with successful closure of the original tunnel orifice, but the new tunnel orifice remained open ( Fig. 1 **d** ). Over the following week, various interventions such as clips, purse-string sutures, and tissue adhesive application three times were employed consecutively ( Fig. 1 **e** ), followed by 10 days of hyperbaric oxygen therapy. On postoperative Day 24, gastroscopy revealed good closure of the new tunnel orifice, and the patient was discharged uneventfully. Follow-up gastroscopy 2 months later confirmed complete wound healing ( Fig. 1 **f** , Video 1 ).

Oxidized regenerated cellulose combined with hyperbaric oxygen therapy successfully managed delayed bleeding in the submucosal tunnel after peroral endoscopic myotomy for achalasia.Video 1

Delayed intratunnel bleeding is a rare complication of POEM. Endoscopic hemostasis measures include re-entry into the tunnel, cleaning up the blood clots, locating the bleeding point, and coagulation, but it is often very difficult. Sengstaken–Blakemore tube compression may achieve hemostasis temporarily but carries complications such as perforation and intolerance 3 . In this case, absorbable oxidized regenerated cellulose coupled with hyperbaric oxygen therapy was successful in managing delayed intratunnel bleeding following POEM. Although the process was tortuous, the result was comfortable.

Endoscopy_UCTN_Code_TTT_1AO_2AD

## Notes

Endoscopy E-Videos https://eref.thieme.de/e-videos
*E-Videos* is an open access online section of the journal *Endoscopy* , reporting on interesting cases and new techniques in gastroenterological endoscopy. All papers include a high-quality video and are published with a Creative Commons CC-BY license. Endoscopy E-Videos qualify for HINARI discounts and waivers and eligibility is automatically checked during the submission process. We grant 100% waivers to articles whose corresponding authors are based in Group A countries and 50% waivers to those who are based in Group B countries as classified by Research4Life (see: https://www.research4life.org/access/eligibility/ ). This section has its own submission website at https://mc.manuscriptcentral.com/e-videos .
